# CD133 expression in different stages of gastric adenocarcinoma

**DOI:** 10.1038/sj.bjc.6605001

**Published:** 2009-04-14

**Authors:** M Boegl, C Prinz

**Affiliations:** 1 Medical Department, Klinikum rechts der Isar, Technical University of Munich, Munich, Germany

**Sir,**

With great interest, we have studied the work by Smith *et al* (2008) that describes presence of CD133 receptors on gastrointestinal cancer cells and states that an antibody-drug conjugate against this receptor led to delayed growth response in gastric cancer cell lines as well as in mice. The work supports the hypothesis that CD133+ cells act as tumorigenic cancer cells.

In reaction to the published work, we have investigated that quantitative CD133 expression in patients with different stages of gastric cancer compared with normal mucosa or chronically inflamed tissue. Interestingly, this expression was reduced in cancer patients and was significantly decreased in patients with more advanced stages of cancer (see Figure 1). These results question the role of CD133 as a marker for tumorigenic gastric cancer cells or question the hypothesis of the potential of such stem cells.

In contrast to the work by Smith *et al* (2008) we determined mRNA levels by RT–PCR, as we were not able to establish a specific antibody binding. This may be because of the scarcity of such tumorigenic cancer cells. In colon cancer, only 2.5% of all cancer cells were found to express CD133 receptors (Ricci-Vitiani *et al*, 2007). If CD133+ cancer cells act as tumorigenic cells although being able to divide, our data question the function of such cells as they were actually reduced in patients with more advanced stages and distant metastasis; in our study, the greatest CD133 expression was found in mucosal stages that are known to have lymph node metastasis only in very rare cases (Gotoda *et al*, 2000).

Alternatively, CD133 receptors may be expressed on other cells than tumour cells. Our data point towards a potential role of CD133 in angiogenesis as VEGF-R2 receptors were closely related to CD133 expression in our collective (correlation coefficient: 0.306; *P*=0.008). Endothelial progenitor cells (EPCs), a subgroup of bone marrow-derived stem cells, were shown to express CD133 receptors together with VEGFR-2, CD34 and VE-cadherin (Peichev *et al*, 2000). Here, CD133 expression was reduced in advanced stages of cancer, paralleling VEGF-R2 expression and microvessel density. Thus, it seems to be that the decrease of CD133 mRNA in advanced stages of gastric cancer reflects reduced angiogenesis and simultaneously reduced presence of endothelial precursor cells.

Overall, the earlier observation of a functional importance of CD133 may be related to the inhibition of angiogenesis and vessel formation, but not directly to a functional role of these receptors in cell proliferation of malignant tumour cells.

## Figures and Tables

**Figure 1:**
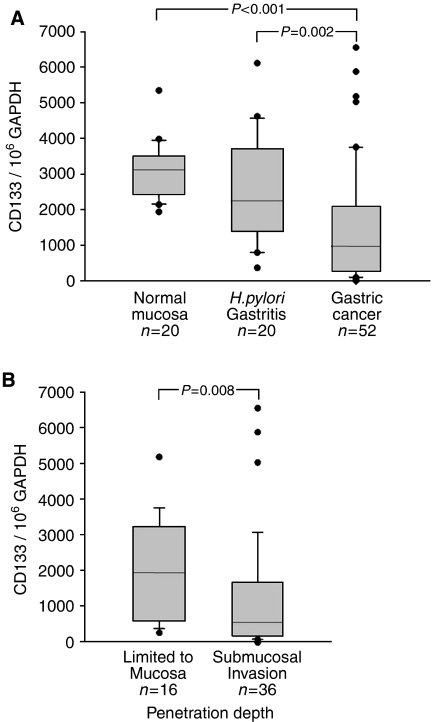
(**A**) CD133 mRNA expression in human gastric tissue samples from 92 patients. mRNA copy numbers were normalised to 10^6^ GAPDH copies. The histological presentations are normal stomach without *H. pylori* infection or inflammation (*n*=20), *H. pylori* infection without severe alterations (*n*=20) and gastric cancer tissue (*n*=52). (**B**) CD133 mRNA expression in human gastric cancer tissue samples from 52 patients. Samples were grouped into carcinomas limited to the mucosa (pT1m) (*n*=16) and carcinomas exceeding the mucosa (pT1sm, pT2, pT3) (*n*=36).
